# Experimental Study on Reinforcement of Reef Limestone by Magnetic Anchoring System

**DOI:** 10.3390/ma16093519

**Published:** 2023-05-04

**Authors:** Jie Liu, Jianxiang Guo, Fan Yu, Zheng Li

**Affiliations:** 1 Key Laboratory of Geological Hazards on Three Gorges Reservoir Area, Ministry of Education, China Three Gorges University, Yichang 443002, China; 2 College of Civil Engineering and Architecture, China Three Gorges University, Yichang 443002, China

**Keywords:** magnetic anchoring system, reef limestone, reinforcement, magnetic anchor

## Abstract

The magnetic anchoring system (MAS) for reef limestone reinforcement is proposed in this paper. The mix proportion of the artificial reef limestone was designed, and the parameters of the MAS were determined through orthogonal tests. The effect of the magnetic field on the anchoring materials was analyzed using XRD and the nitrogen adsorption method. The results indicate that the designed artificial reef limestone can be used in place of in situ rock samples for laboratory tests. In air, the bond samples of the anchoring material and reef limestone experienced cohesion failure of the artificial reef limestone. However, in seawater, it was cohesion failure of the reef limestone and interface adhesion failure. During the pull-out test, the reef limestone specimen reinforced by MAS showed interface failure between the anchoring material and the rock mass. The Fe_3_O_4_ powder present in the anchoring material has the ability to migrate towards the anchor, thereby enhancing the density of the anchoring material. This, in turn, helps to eliminate the free water present in the anchor hole, and consequently, improves the bonding effect of the interface. The reinforcement effect of MAS is particularly advantageous for rock reinforcement under complex working conditions.

## 1. Introduction

Reef limestone commonly used in marine engineering, as a geotechnical medium, is primarily formed through the deposition of marine biological remains. It is also referred to as biological skeleton limestone [1,2,3]. Reef limestone is primarily found in the tropical marine area between north and south latitudes [4,5]. Reef limestone diagenesis is primarily caused by biochemical cementation, gravity compaction, and late cold metamorphism [5,6,7]. Reef limestone is primarily composed of calcite, aragonite, and dolomite minerals, with a predominant chemical composition of calcium carbonate [5]. Reef limestone is generally considered as soft rock, with irregular shape, multi-angle, high porosity, fragile characteristics [8,9,10]. As the marine economy continues to grow and marine protection becomes increasingly important, finding effective ways to reinforce reef limestone in marine engineering construction while reducing environmental pollution has become an urgent priority.

The epoxy-bonded anchor system (EBAS) is a common technical measure for underwater rock reinforcement [11,12,13]. The reinforcement effect is significantly impacted by the performance of epoxy resin when used as an anchorage material [14]. As a result, researchers have conducted studies on modifying epoxy resin to improve its effectiveness. Boumaza et al. [15] observed that the addition of micro- and nano-sized fillers can improve the elastic modulus and hardness of the epoxy resin. It was found that SiO_2_ microparticles were superior to micro Al_2_O_3_ and micro TiO_2_ in improving the mechanical properties of epoxy resin [16]. Dorigato and Pegoretti [17] discovered that the addition of Al_2_O_3_ nanoparticles can enhance the adhesion property of epoxy resin. Bagherzadeh [18] incorporated 600 mesh silica into epoxy resin, resulting in enhanced mechanical properties of the resin. Ahmad et al. [19] reported that mica microparticles provided higher pull-out strength than nano silica in modified epoxy resin. Many achievements and progress have been made by previous research in epoxy-bonded anchor systems. However, in the current grouting process, some of the anchoring materials will inevitably gush out from the gap between the anchor plate and the bedrock, which is neither economical nor environmentally friendly. Developing an effective and environmentally friendly underwater rock reinforcement technology remains a challenge due to the difficulty in completely discharging water from anchor holes during grouting, which can diminish the anchoring effect.

Magnetic fluid is a novel functional material that exhibits liquid mobility and magnetism, allowing it to flow under the influence of a magnetic force. It has been widely used in the fields of sealing and shock absorption under various harsh conditions [20,21]. Inspired by the concept of magnetic fluid, some scholars have explored the optimization of steel fiber arrangement in steel fiber cement-based composites by electromagnetics [22,23,24]. Chen et al. [25] and Xue et al. [26] conducted a study where they substituted concrete aggregate with steel slag and utilized an external magnetic field to induce the deflection and motion of steel slag in order to vibrate the concrete.

**Figure 1:**
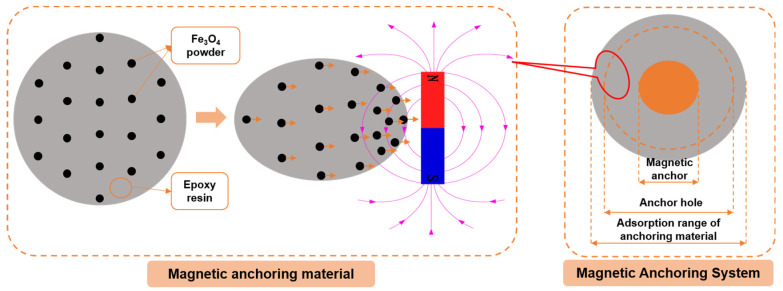
Concept diagram of MAS.

Referring to this idea, this paper proposes a new magnetic anchoring system that utilizes Fe_3_O_4_ power as the anchoring material and a magnetic metal rod as the anchor bolt (as shown in Figure 1). The mix proportion of artificial reef limestone was designed, and the parameters of the magnetic anchoring system were determined through orthogonal tests. Then, the bond performance between the anchoring material and reef limestone was analyzed by the direct shear test. Next, the reinforcement effect of reef limestone by the magnetic anchoring system was evaluated by a pull-out test. Last, the effect of the magnetic field on the internal microstructure of the anchoring materials in MAS was analyzed by the XRD and nitrogen adsorption method.

## 2. Materials and Methods

### 2.1. Preparation of Artificial Reef Limestone

**Figure 2:**
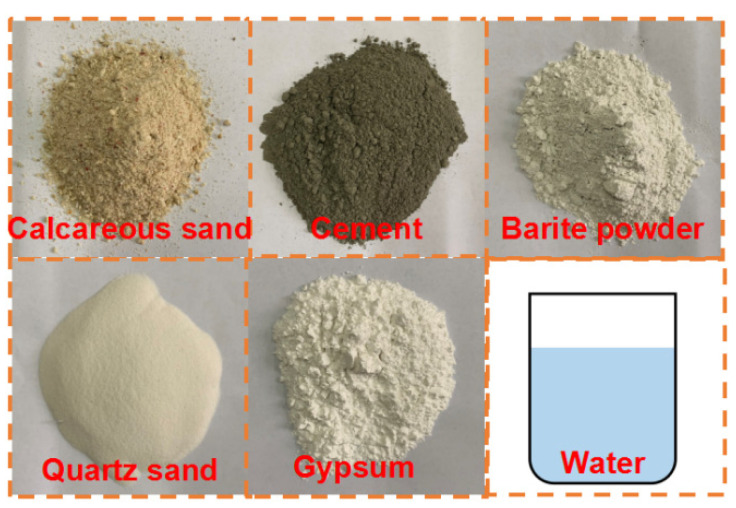
Mix design of artificial reef limestone.

Reef limestone belongs to the category of soft rock, making it challenging to acquire large in situ rock samples. Furthermore, due to the unique region and origin of this type of limestone, the current research objects available for indoor study are limited to small in situ rock samples. Therefore, this study aims to create artificial reef limestone rock samples using the raw materials listed in Table 1 (see Figure 2). According to the existing research results [27], the mix proportion of artificial reef limestone includes 30% barite powder and 10% calcareous sand of the total mass. The amount of other raw materials, such as quartz sand, gypsum, cement, and water, was determined through an orthogonal test. The design of this test is shown in Table 2 and Table 3. The optimal ratio of artificial reef limestone was evaluated through bulk density test, uniaxial compression test, and indirect tensile test results. By uniaxial compression test, indirect tensile test, and direct shear test, the mechanical parameters of the optimal ratio of artificial reef limestone were obtained and compared with the original reef limestone sample.

### 2.2. Design of Epoxy-Magnetic Powder-Magnetic Anchor System

**Figure 3:**
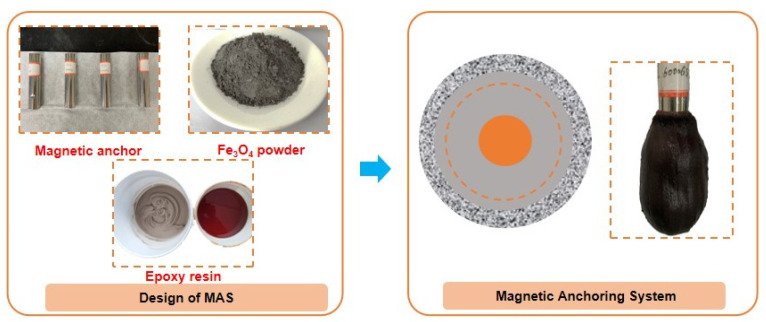
Epoxy-Magnetic Powder-Magnetic Anchor System.

The material composition of the epoxy-magnetic powder-magnetic anchor system (magnetic anchoring system, MAS) proposed in this study includes epoxy, Fe_3_O_4_ powder, and a magnetic anchor, as shown in Figure 3. The properties are listed in Table 4. The magnetic anchor rod of the MAS can absorb and aggregate the anchoring material, resulting in a more compact and solid interface between anchors. Therefore, it is necessary to ensure that the absorption range of the anchoring material is always wider than the diameter of the anchor hole. The absorption range of the anchoring material is influenced by several factors, including the viscosity of the anchoring material, the strength of the magnetic field, and the diameter of the anchor rod. The viscosity of the anchoring material is affected by the content of the Fe_3_O_4_ powder. Therefore, three factors and four levels of epoxy/Fe_3_O_4_ powder ratio, magnetic field intensity, and magnetic rod diameter were established for the orthogonal test. Table 5 shows the factor levels, while Table 6 displays the orthogonal test table.

Fe_3_O_4_ powder was mixed with epoxy A adhesive and stirred for 3 min. Then, epoxy B adhesive (half the amount of epoxy A adhesive) and a diluent (5% of the total epoxy mass) were added and stirred for an additional 5 min. Last, the anchorage material mixture was poured into the mold, and the mold was removed after curing for 1 day.

### 2.3. Test Method

#### 2.3.1. Absorption Range of the Anchoring Material

**Figure 4:**
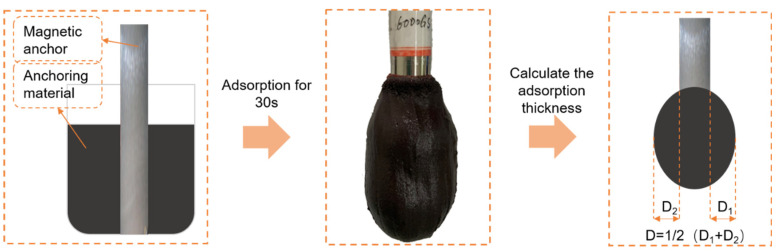
The method of measuring the absorption diameter of the anchoring material.

The filling density of anchoring in the anchor hole is determined by the absorption range of the anchoring material. To evaluate the absorption range, the index of absorption diameter (D) was used in this study. First, a mixture of anchoring material totaling 1.5 L was prepared and placed into a 2 L beaker. Second, a magnetic bolt was inserted into the center of the beaker and removed after 30 s of adsorption. A photograph was taken, and the adsorption diameter was measured, as shown in Figure 4.

#### 2.3.2. Mechanical Property

(1) Uniaxial compression test

**Figure 5:**
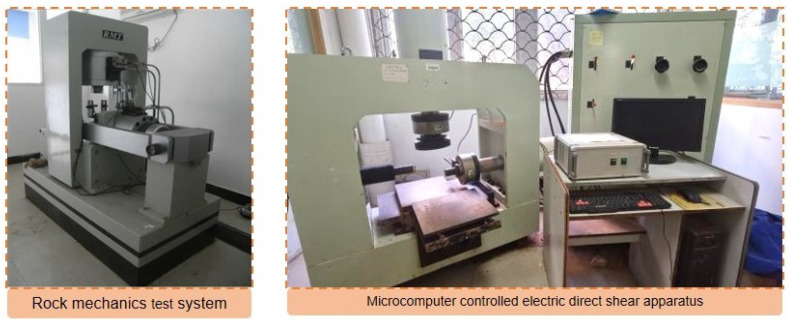
Testing instrument for mechanical properties.

A rock mechanics test system (Figure 5) was used to test a cylinder with dimensions of 100 mm × *φ*50 mm. The loading process was displacement control, with a rate of 0.005 mm/s and a maximum displacement limit of 4.2 mm.

(2) Indirect tensile test

The test specimen was a cylinder with dimensions of 50 mm in diameter and 50 mm in height. The rock mechanics test system (Figure 5) was used as the testing instrument. The loading process was controlled by stroke at a rate of 0.005 mm/s, with a maximum stroke limit of 4.7 mm.

(3) Direct shear test

The study utilized a 100 mm × 100 mm × 100 mm cube as the sample for testing. The test instrument used was a microcomputer controlled electric direct shear apparatus, as shown in Figure 5. The specimen was subjected to a specific normal stress during the test. After a 15 s stabilization period of the normal force, the tangential force was applied using a displacement control process. The shear rate was fixed at 0.5 mm/min and the endpoint for the shear displacement was set to 15 mm.

#### 2.3.3. Pull-Out Test

(1) Samples preparation

**Figure 6:**
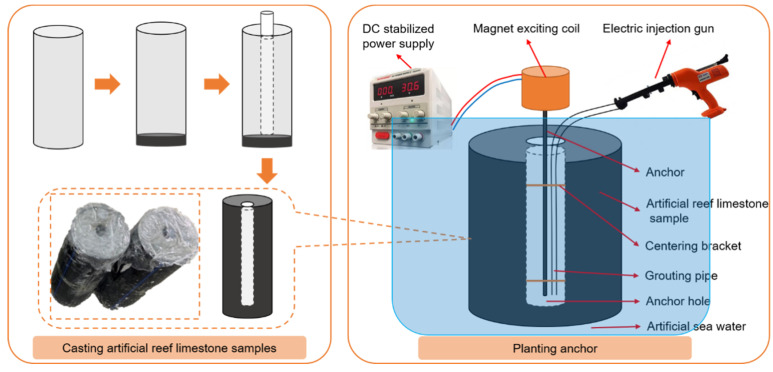
Preparation of test samples.

The PE pipe with outer diameter 200 mm, inner diameter 170 mm, height 500 mm, and bottom sealing was selected as the model. First, 50 mm of artificial reef limestone mixture was poured into the test mold. After 1 d of curing, a PVC pipe with a length of 600 mm was placed in the center of the mold; the outer diameter was determined according to the diameter of the anchor hole. To create the artificial reef limestone samples with anchor holes, the area between the test mold and PVC pipe was filled with the reef limestone mixture. After 20 min, the PVC pipe was removed, leaving behind the desired samples. After three days of spraying and curing, the samples were immersed in artificial seawater for further curing. The anchor was secured in place using a centering bracket and the grouting pipe was fixed between the anchor and the wall of the anchor hole. Finally, the electric injection gun was connected to the grouting pipe. The free end of the anchor was inserted into the magnet excitation coil and fixed, and the magnet excitation coil was connected with the DC-stabilized power supply. The power supply was adjusted to the designed voltage and current to magnetize the anchor. Then, the anchoring material was slowly injected into the anchor hole. After injecting, the voltage (24 V) should be maintained for 5 min before being reduced to 5 V. Once this is done, the power supply should be disconnected, and the magnet excitation coil should be removed after 1 h. The entire planting anchor process should take place under the immersion of artificial seawater. The sample preparation process is shown in Figure 6.

(2) Test procedure

**Figure 7:**
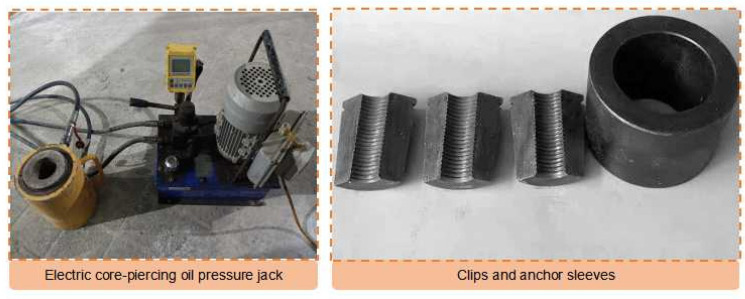
Pull-out test equipment.

The servo loading system for the pull-out test was the electric piercing oil pressure jack (shown in Figure 7), which can automatically record the load and displacement and provide a maximum tensile force of 500 kN. The loading speed was 0.5 mm/min.

## 3. Results and Discussions

### 3.1. Mix Design of Artificial Reef Limestone

**Figure 8:**
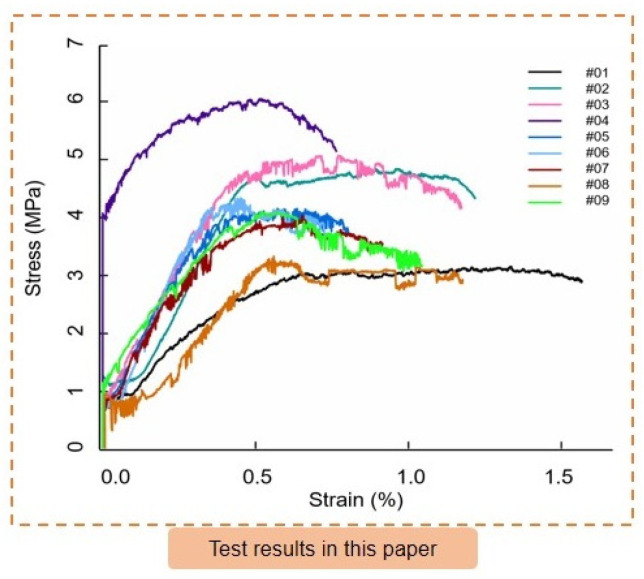
Stress-strain curves of artificial reef limestone samples.

Figure 8 displays the uniaxial compressive stress-strain curves for the artificial reef limestone samples in groups 1–9. It can be seen from the figure that the residual stress after the peak of the stress-strain curve is large, and even some curves have no obvious peak, which is similar to the results of the original reef limestone sample studied by Zhu et al. [28]. The maximum peak strength of the artificial reef limestone is 1.81 times greater than the minimum peak strength. On the stress-strain curve, the stress gradually drops after the peak. Even the first and eighth groups exhibit obvious stress yield platforms, indicating a transition towards ductile failure. This suggests that the failure mode of the artificial reef limestone has gradually changed to a ductile failure type. Compared to sedimentary rocks on land, artificial reef limestones exhibit greater axial deformation and demonstrate typical compressibility.

**Figure 9:**
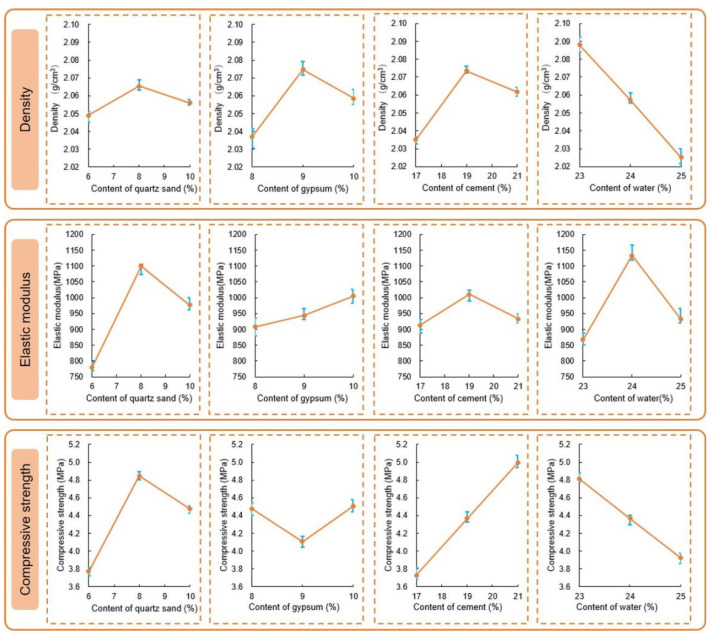
The range analysis of the orthogonal test results.

Table 7 and Figure 9 present the range analysis of the orthogonal test results. The results show that the water content is the most sensitive factor affecting the material density of artificial reef limestone. As the water content increases, the material density decreases. On the other hand, quartz sand has little effect on density. In terms of the sensitivity descending order of the elastic modulus to the factors, quartz sand has the highest sensitivity followed by water, cement, and gypsum. The density and elastic modulus of the nine groups of artificial reef limestone materials are in the range of corresponding index distribution of reef limestone original rock. The sensitivity of the uniaxial compressive strength to the factors was observed to follow the descending order of cement > quartz sand > water > gypsum. The uniaxial compressive strength of group 4 was found to be closest to the maximum distribution range (6~9 MPa) of the original reef limestone. Furthermore, it was evident that the strength of group 4 was higher than the other eight groups studied. This paper presents the design principle of the artificial with the aim of achieving high uniaxial compressive strength. The study determined the optimal level combination of each factor, namely quartz sand, gypsum, cement, and water, which was found to be 2, 3, 3, and 3, respectively.

The uniaxial compression, indirect tensile, and direct shear tests were conducted on the artificial reef limestone material under the optimal ratio to obtain its physical and mechanical parameters, which were compared with the original reef limestone sample. The results are exhibited in Table 8. Based on the test results, it was found that the mechanical indexes of the artificial reef limestone were within the range of the indexes of the original reef limestone. This indicates the artificial reef limestone made in this study can be used as a suitable replacement for in situ rock samples in indoor test research.

### 3.2. Design of MAS

**Figure 10:**
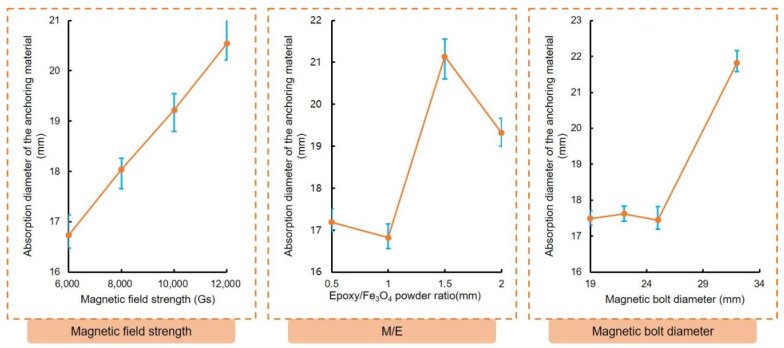
The range analysis result of the absorption range of the anchoring material.

The absorption range of the anchoring material was analyzed and results are depicted in Figure 10. It was observed that the sensitivity of the absorption range to the factors followed a descending order of magnetic bolt diameter, epoxy/Fe_3_O_4_ powder ratio, and magnetic field intensity. The thickness of the absorption range in the figure for the anchoring material is greater than 16 mm, which surpasses the required 3~6 mm for the epoxy-bonded anchor system. The test revealed that if the ratio of epoxy to Fe_3_O_4_ powder is too high, the viscosity of the anchor material becomes too thick and hinders construction. Conversely, if the ratio is too low, the material segregates. Therefore, to ensure a better grouting effect, a ratio of 1:1 epoxy/Fe_3_O_4_ powder was utilized in the preparation of the anchoring material for this study.

According to Figure 9, there is a positive linear correlation between the magnetic field strength and the adsorption range. However, the current anchor bolt magnetization method has limitations and can only reach up to 6000 Gs. During the pull-out test, the magnetic field strength used was below 6000 Gs. It is important to note that the magnetic field strength and adsorption range have a linear relationship. Even when the magnetic field intensity decreased to 2000 Gs, the adsorption range remained significant at 14 mm, which is more than 3~6 mm. The study found that the diameter of the magnetic bolt had little influence on the adsorption range when it was within 19~25 mm. However, when the diameter reached 32 mm, the adsorption range significantly increased. Therefore, for the pull-out test, two diameters (19 mm and 25 mm) were selected to test the anchor bolt. Assuming an adsorption range of 14 mm, the diameter of the MAS should be between 47 mm and 53 mm. As a result, the diameter of the anchor hole in the pull-out test can be set to 40 mm or 50 mm.

**Figure 11:**
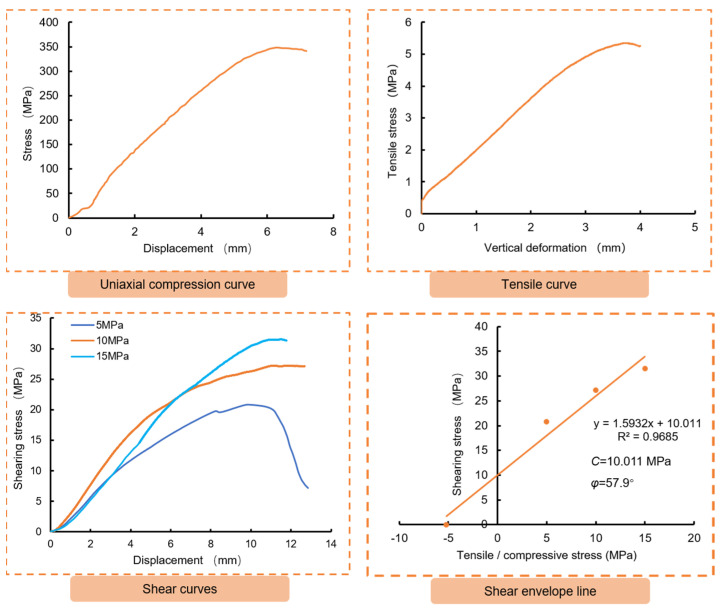
The test results of the anchoring material.

**Figure 12:**
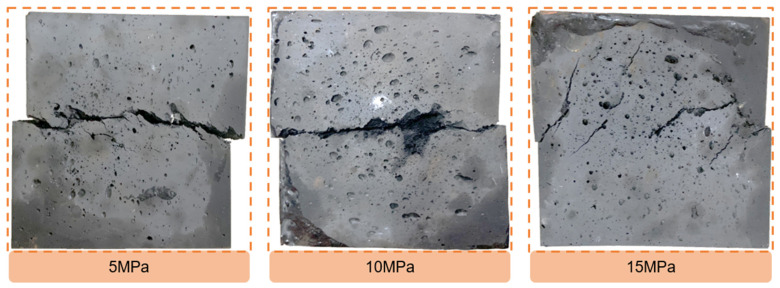
Failure samples after direct shear test.

The study conducted uniaxial compression, indirect tensile, and direct shear tests on anchoring material with an epoxy/Fe_3_O_4_ powder ratio of 1:1. The results of these tests are presented in Figure 11 and Figure 12, while the physical and mechanical properties of the anchoring material are summarized in Table 9. The obtained curves from all three tests in Figure 10 showed significant strain softening, with a large displacement at the peak. The damaged samples in Figure 12 indicate that almost all of the cracks were not fully repaired. This is consistent with the property of the epoxy resin itself, suggesting that the addition of Fe_3_O_4_ powder did not significantly affect the deformation characteristics of the epoxy resin. It can be seen from Table 9 that the mechanical properties of the anchoring material were significantly lower than those of the epoxy resin, but they were still significantly higher than those of the anchoring object (artificial reef limestone), so as to avoid the damage of the anchor structure from the anchoring material. Therefore, it can be considered that the magnetic anchoring material designed in this paper can be used for reef limestone reinforcement.

### 3.3. Bond Performance between Anchoring Material and Reef Limestone

**Figure 13:**
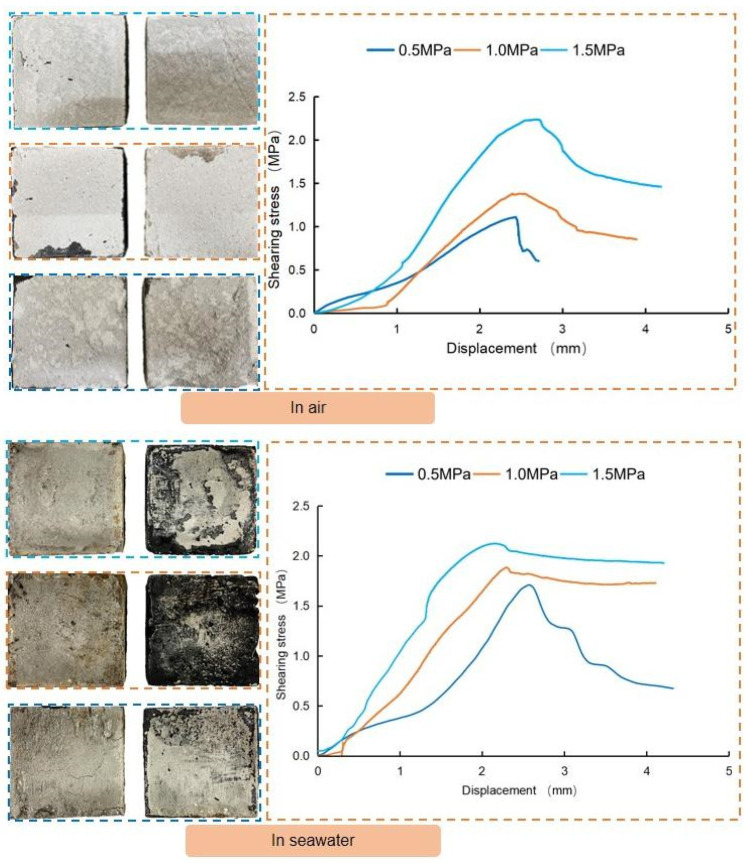
Direct shear test results of the bond block sample.

In order to investigate the bonding performance between the anchoring material and artificial reef limestone, bond block samples of the two materials were prepared and subjected to direct shear tests under two different environmental conditions: air and seawater. Figure 13 displays the results of these tests. The results show that the failure mode of the samples tested in air was cohesion failure of artificial reef limestone, whereas the samples tested in seawater showed cohesion failure of reef limestone and interface adhesion failure. It indicates that the damage of the anchoring material (designed in this paper) itself will not occur when it is used to reinforce the reef limestone.

Another interesting finding is that when the vertical pressure was low (0.5 MPa, 1.0 MPa), the peak shear stress of the samples in seawater was significantly higher than in air. However, as the vertical pressure increased to 1.5 MPa, the gap between the two decreased significantly. This can be explained by the fact that at low vertical stress, the shear stress required to destroy the samples in seawater is greater than the resultant force of the bonding force and the water film tension. Whereas, as the vertical pressure increased, the seawater was extruded from the interface and the pore channels of the reef limestone. This led to a significant reduction in water film tension.

### 3.4. Results of Pull-Out Test

**Figure 14:**
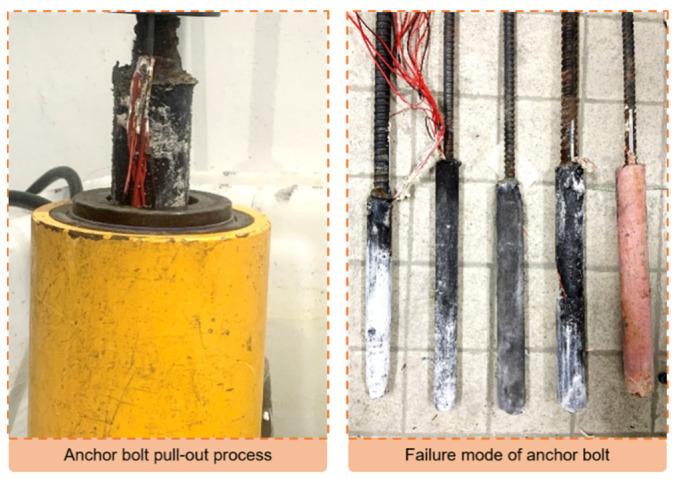
Process and failure mode of pull-out test.

**Figure 15:**
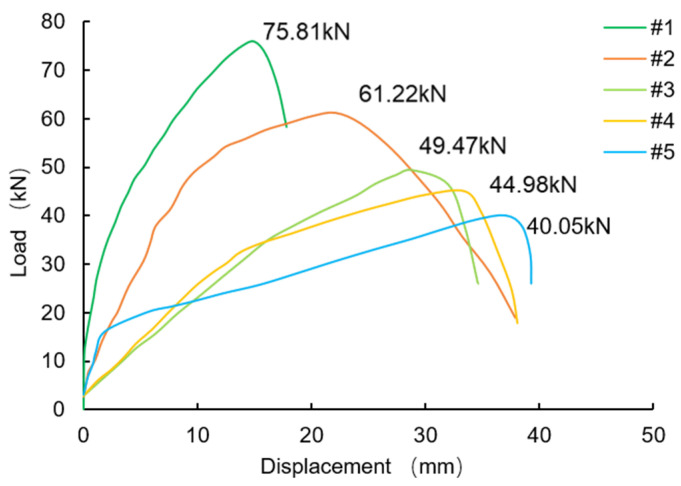
Load-displacement curve of pull-out test.

The orthogonal test of two levels was conducted with the anchor bolt diameter, anchor hole diameter, and magnetic field strength as three factors, and a control group was set up. The test scheme is shown in Table 10. The pull-out test process and the pulled-out bolt are shown in Figure 14. The study found that the reef limestone specimen reinforced by the bolt failed at the interface between the anchoring material and the rock mass, which aligns with the failure mode observed in the direct shear test. The results of the pull-out test are presented in Figure 15. Additionally, an analysis of the orthogonal test results revealed that the range of the anchor bolt diameter, magnetic field strength, and anchor hole diameter were 21.29, 9.54, and 5.05, respectively. The study found that the sensitivity of the load peak to the factors decreased in the following order: anchor bolt diameter, magnetic field intensity, anchor hole diameter. The optimal combination of factors for achieving the ultimate pullout force was determined to be an anchor bolt diameter of 25 mm, magnetic field strength of 4000 Gs, and anchor hole diameter of 50 mm (1,1,1), with group #1 being the most optimal.

In comparison to the traditional anchoring methods (#4 and #5), although the mechanical properties of the anchoring material in the MAS were significantly reduced when compared to the resin, the peak load of the pull test was still 12.3% higher. This is due to the magnetic anchor bolt in the MAS, which has an evident absorption effect on the anchoring material. This feature has the potential to improve the density of the anchoring material, which in turn can help to remove any free water present in the anchor hole. The process can enhance the bonding effect of the interface. In comparison to #3 and #5, increasing the magnetic field strength can continue to improve the anchoring effect of the MAS, indicating that the reinforcement effect of the MAS has the potential to be further improved. Moreover, the MAS has an absorption effect on the anchorage materials, which gains more advantages for rock reinforcement under complex working conditions (anti-dip angle).

### 3.5. Micro Analysis

**Figure 16:**
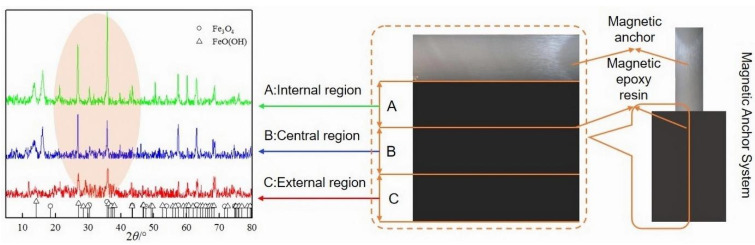
Results of XRD.

This study aimed to investigate the impact of magnetic fields on the internal microstructure of anchoring materials in the MAS. The anchoring material was categorized into three regions (A, B, C) according to the distance between the anchoring material and the anchor bolt. The mineral composition of the anchor material was measured by X-ray diffraction (XRD), and the results are shown in Figure 16. It can be seen from Figure 16 that with the increase in the distance between the anchoring material and the bolt, the heights of the characteristic peaks of Fe_3_O_4_ (PDF-#26-1136) and FeO(OH) (PDF-#22-0353) gradually increased. In the mixture of resin and Fe_3_O_4_ powder (anchoring material), these two minerals can only be brought by Fe_3_O_4_ powder, indicating that under the attraction of magnetic force, Fe_3_O_4_ powder will migrate to the anchor. Based on the diffraction peak intensity of the two minerals in the three regions, it can be concluded that the magnetic powder was not completely concentrated near the anchor bolt. The concentration of magnetic powder near the anchor is the largest, and the concentration decreases with the increase in distance from the anchor bolt.

**Figure 17:**
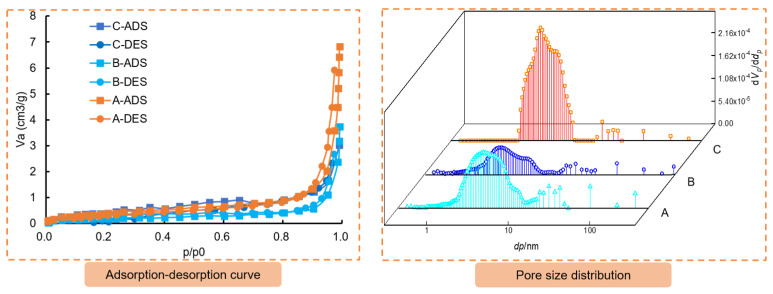
Adsorption-desorption curves and pore size distribution of the anchoring material.

The pore structure was analyzed using the nitrogen adsorption method, with the results presented in Figure 17. The adsorption-desorption isotherms of the anchoring materials in each region were classified according to the IUPAC isotherm standard [29] and found to belong to type II (S-type isotherm). This type of isotherm represents a free single multilayer reversible adsorption process. Table 11 shows the data for BET surface area and total pore volume for the anchoring materials of A, B, C. The study found that the average pore diameter of each region of the anchoring material belongs to mesoporous (2~50 nm). The pore diameter of region A and B are mostly within the micropore (<2 nm) and mesoporous range. Region C, however, contains lots of mesoporous and very few large pores (>50 nm). It can be speculated that during grouting, more mesoporous near the anchor were compressed or separated into micropores under the attraction of the magnetic field force.

## 4. Conclusions

In this paper, the conception of the MAS used for reef limestone reinforcement was proposed and designed by an orthogonal test. The mix proportion of artificial reef limestone was designed. The reinforcement effect of reef limestone by the MAS was evaluated by direct shear test and pull-out test. The effect of the magnetic field on the internal microstructure of the anchoring materials in the MAS was analyzed by XRD and the nitrogen adsorption method. The main conclusions are as follows:

(1) The orthogonal test of the relative ratio of the reef limestone was carried out. The concept of a magnetic anchorage system (MAS) in reef limestone was proposed.
(2) In the pull-out test, it was observed that the interface failure between the anchoring material and the rock mass occurred in the reef limestone specimen that was reinforced by the bolt. The peak load of the pull test was 12.3% higher than that of the traditional anchoring method. The feature that the magnetic anchor bolt adsorbing the anchoring material can improve the density of the anchoring material, and squeeze out the free water in the anchor hole, improved the bonding effect of the interface.
(3) Under the attraction of magnetic force, the Fe_3_O_4_ powder migrated to the anchor (but not all concentrated near the anchor). Additionally, the mesoporous near the anchor was extruded and separated into microspores, so as to improve the compactness of the anchoring material.

## Figures and Tables

**Table 1:** Raw materials of artificial reef limestone.

Calcareous Sand	Cement	Barite Powder	Quartz Sand	Gypsum Powder
Original calcareous sand from south China sea.	Portland cement; NO.42.5.	White powder; Diameter: 20 μm.	White translucent; Diameter: 0.45~0.2 mm.	White powder; Diameter: 9 μm.

**Table 2:** Orthogonal test factor level table of artificial reef limestone (%).

Level	Quartz Sand	Gypsum	Cement	Water
1	10	8	17	25
2	8	9	19	24
3	6	10	21	23

**Table 3:** Orthogonal test table of artificial reef limestone.

Code	Quartz Sand	Gypsum	Cement	Water
1	1	1	1	1
2	1	2	3	2
3	1	3	2	3
4	2	1	3	3
5	2	2	2	1
6	2	3	1	2
7	3	1	2	2
8	3	2	1	3
9	3	3	3	1

**Table 4:** Properties of MAS components.

Epoxy	Fe_3_O_4_ Powder	Diluent	Magnetic Anchor
Density:1.50 g/cm^3^; Viscosity:20 Pa·s; Solid content: 99.5%; Compressive strength: 92.9 MPa; Bending strength: 61.7 MPa; Split tensile strength: 17.7 MPa.	Diameter: 1~3 μm.	98° Industrial alcohol.	Material: NdFeB cylindrical magnet; Diameter: 19~32 mm; Magnetic field intensity: 6000~12,000 Gs.

**Table 5:** Orthogonal test factor level table of MAS.

Level	Magnetic Field Intensity (Gs)	Epoxy/Fe_3_O_4_ Powder Ratio	Diameter of Magnetic Anchor (mm)
1	6000	2	19
2	8000	1	22
3	10,000	1/1.5	25
4	12,000	0.5	32

**Table 6:** Orthogonal test table of MAS.

Code	Magnetic Field Intensity	Epoxy/Fe_3_O_4_ Powder Ratio	Diameter of Magnetic Anchor
1	1	1	1
2	1	2	2
3	1	3	3
4	1	4	4
5	2	1	2
6	2	2	1
7	2	3	4
8	2	4	3
9	3	1	3
10	3	2	4
11	3	3	1
12	3	4	2
13	4	1	4
14	4	2	3
15	4	3	2
16	4	4	1

**Table 7:** Orthogonal test results of artificial reef limestone mixture.

Code	Density (N·m^−3^)	Compressive Strength (MPa)	Elastic Modulus (MPa)
1	19.83	3.51	797.50
2	20.79	4.84	1130.08
3	21.06	5.07	1002.30
4	20.82	6.04	950.91
5	20.69	4.15	1053.96
6	20.46	4.34	1295.49
7	20.46	3.88	974.80
8	20.76	3.3291	647.738
9	20.24	4.0991	718.489

**Table 8:** Comparison of performance parameters of artificial reef limestone with original sample.

	Compressive Strength (MPa)	Tensile Strength (MPa)	Elastic Modulus (GPa)
Artificial reef limestone	6.70	0.66	1.20
Original sample [8,10]	1.07~38.60, (most of them are 6~9)	0.64~4.4	0.61~14.08

**Table 9:** Physical and mechanical properties of the anchoring materials.

Volumetric Weight (N·m^−3^)	Compressive Strength (MPa)	Tensile Strength (MPa)	Elastic Modulus (MPa)	Poisson Ratio	Cohesion (MPa)	Angle of Internal Friction (°)
21.02	36.31	5.33	393.60	0.19	10.01	57.9

**Table 10:** Pull out the test scheme.

Code		Anchor Bolt Diameter	Magnetic Field Strength	Anchor Hole Diameter	Anchoring Material
Orthogonal test	#1	1; (25 mm)	1; (4000 GS)	1; (50 mm)	Epoxy + Fe_3_O_4_ powder
	#2	1; (25 mm)	2; (2000 GS)	2; (40 mm)	
	#3	2; (19 mm)	1; (4000 GS)	2; (40 mm)	
	#4	2; (19 mm)	2; (2000 GS)	1; (50 mm)	
Control group	#5	19 mm	——	50 mm	Epoxy

**Table 11:** BET surface area and total pore volume.

Region	BET Surface Area (m^2^·g^−1^)	Total Pore Volume (cm^3^(STP) g^−1^)
A	0.66506	0.1528
B	1.4494	0.333
C	1.6831	0.3867

## Data Availability

The research data are included within the article, and further data are available from the first and corresponding author upon request.
